# Caring for the person with cancer: Information and support needs and the role of technology

**DOI:** 10.1002/pon.4722

**Published:** 2018-04-20

**Authors:** Natalie Heynsbergh, Mari Botti, Leila Heckel, Patricia M. Livingston

**Affiliations:** ^1^ Faculty of Health, School of Nursing and Midwifery Deakin University Geelong Victoria Australia; ^2^ Epworth HealthCare Victoria Australia; ^3^ Faculty of Health, Deakin University Geelong Victoria Australia

**Keywords:** cancer, caregivers, communication, information, needs assessment, neoplasms, oncology, technology

## Abstract

**Objective:**

Informal carers experience a variety of information and support needs when providing care to someone with cancer. It is unclear when carers seek information and what resources they access to support themselves throughout the cancer trajectory.

**Methods:**

A sample of 45 carers and 15 oncology nurses were recruited to participate in either focus groups or phone interviews.

**Results:**

Carers in the study were more likely to be women (60%), caring for a spouse or partner (64.4%), living with the patient (86.7%), and hold a university degree (46.7%). The majority of oncology nurses were females (66.6%). Findings showed that carers had limited access to adequate information as needs arose. Supports used to address information needs included information booklets, the Internet, and communication with healthcare professionals or with other carers. Barriers in communication between nurses and carers impacted on the adequacy of information received. Participants reported that technology, such as smartphone applications, might be appropriate for improving information and support needs**.**

**Conclusions:**

Caring for someone with cancer is multifaceted. Carers need access to timely information to help them effectively manage patients' needs. Future studies should assess the role of contemporary approaches, such as digital technology, as a solution to the delivery of information and support for carers of people with cancer.

## 1 INTRODUCTION

Cancer is a significant public health issue worldwide with 14 million people diagnosed with cancer in 2012.^1^ In Australia, it was estimated that over 130 000 people were diagnosed with cancer in 2017.^2^ Developments in cancer screening initiatives and treatment mean that many more people are receiving treatment earlier and, in 2012, 68% of people with cancer reached 5‐year survivorship.^2^ Approximately 1 in 10 hospital admissions is cancer related, and between 2008 and 2009, $4526 million of the healthcare budget was spent on cancer.^2^ The financial implications on the healthcare system of cancer‐related admissions have resulted in earlier patient discharge from hospital,^3^ which when considered with the increase in survivorship of cancer^4^ has meant a shift from care provided in hospitals to informal care provided in the home. As a consequence, there is an increasing requirement for informal carers in the community. Carers perform a multitude of duties including meeting physical and emotional needs, medication administration, providing transport, and attending medical appointments.^5^ These carers are known as “informal carers” as they are not paid for the care they provide and often lack access to information, skills, and resources necessary to provide holistic care to the person with cancer.^6^

The information needs of carers have been assessed extensively in the literature. It is common for carers to have unmet needs related to patients' diagnosis, prognosis, physical and psychological impacts of treatment, and how to manage care needs at home.^7^, ^8^ While information needs decrease over time, some carers still experience unmet information needs throughout treatment and at the end of treatment.^9^ Carers have used a variety of sources to address their unmet needs, most commonly relying on healthcare professionals, family, friends, and the internet.^10^ However, current methods of delivering information are often inadequate. Oberoi et al^8^ for example found that out of 196 carers, only 33% were certain that they received information on managing patients' symptoms and only 18% were asked by nursing staff if they required more support.

What we know little about is the most efficacious way to deliver information and how support can be provided using more contemporary technology‐based approaches. This analysis was undertaken as part of a study in which the overall aim was to explore informal carers' experience**s** of caring for people with cancer in their own homes, their information needs, adequacy of knowledge received, and the support and services carers used to address their needs, their perceived barriers to accessing support services, and how technology may address carers' needs.

The focus of the analyses reported in this paper was on the information and support sought by carers and how technology may address needs identified. Findings from this paper will inform the development of a smartphone application intervention to address carers' needs.

### 1.1 Research questions:

1. What information and communication support mechanisms were sought by carers when meeting the needs of patients with cancer?
2. How can technology overcome perceived barriers to accessing information and support?

## 2 METHODS

### 2.1 Design

A qualitative descriptive approach was applied^11^ to explore carers' and oncology nurses' perspectives on information and communication support needs of carers. This approach allowed for open‐ended questions to facilitate a detailed understanding of the topic, permitted concepts to be explored from the participants' unique situations, and themes to be developed from common concepts in data.^11^ Data were collected via focus groups or phone interviews depending on participant availability. Ethics approval was obtained from Deakin University Human Research Ethics Committee (HREC): 2016‐118 Eastern Health HREC: LR 136‐2015 and Epworth HealthCare HREC: 715‐15.

### 2.2 Sample

Recruitment continued until saturation of data occurred. Forty‐five carers and 15 oncology nurses were recruited from a large public health service, a private health service, and a carer organisation. Oncology nurses included in the study had completed training in administering and managing the care needs of patients receiving chemotherapy and were providing care to patients of carers who participated in the study. All participants were informed that this research was conducted as part of a PhD project and provided written informed consent.

#### 2.2.1 Procedure

Carers were approached directly during patients' outpatient chemotherapy appointments or via a carer organisation through email invitations sent by organisation personnel. Oncology nurses were recruited during staff meetings at the participating health services. Participants were given the opportunity to attend 1 of 2 focus groups or, if unavailable, to participate in a phone interview.

#### 2.2.2 Inclusion criteria

Informal carers were current or recent previous carers of people with cancer (referred to as “the patient”) receiving active or palliative treatment. Oncology nurses were working in direct contact with patients and carers over their treatment trajectory in day oncology units.

#### 2.2.3 Exclusion criteria

Carers aged under 18 years and those with a non‐English speaking background who would require interpreters to participate in the study.

## 3 DATA COLLECTION

Semistructured questions guided focus group and individual interviews to explore the experiences of carers and oncology nurses in communication of information. Focus groups were separated into carer only and oncology nurse only groups. Phone interviews and focus groups were conducted between May and June 2016. The phone interviews were conducted by 1 researcher (NH), and the focus groups were facilitated by all the researchers. Participants in focus groups and phone interviews were asked the same questions. Carers were asked to describe their experiences, unmet needs, support systems used, and how technology could be incorporated to address their needs. Oncology nurses were asked to explore their perceptions of carers' experiences, challenges, and support services they provided or that were available to carers. Prompts were used to clarify questions and to focus participants on the topics relevant to the research in both the phone interviews and focus groups. Focus groups were conducted within the carer organisation or health facilities but in rooms separate from day oncology units. Phone interviews were conducted when the researcher was in a private room and carers were in a location where they felt comfortable. Field notes were collected during or immediately after focus groups. All interviews and focus groups were audio‐recorded and transcribed in full. The list of questions used in focus groups and phone interviews is outlined in Table 1.

**Table 1:** Focus group and interview questions

Carers	Nurses
Describe your experiences as a carer of a person with cancer. How would you describe the challenges of being an informal carer? Have you accessed any support services? Thinking back to when your family member/friend first began treatment, what do you know now that you would have liked to have known then? How have the health services supported you during this time? How could digital technology such as smartphone apps support you while providing care to someone with cancer?	How would you describe informal carers' experiences when caring for a person with cancer? How would you describe the challenges of being an informal carer? What external support services do informal carers access? What services/websites do you recommend to carers? Why do you recommend these resources? How could digital technology such as smartphone apps support carers in their role?

Demographic characteristics collected from carers included age, gender, education, relationship to the person with cancer, and living situation.

## 4 DATA ANALYSIS

Audio‐recorded data were transcribed verbatim and were coded manually. Coded data were initially organised by separating transcript excerpts verbatim into broad topics with subsequent subtopics that were further organised using a numbering system (coded and organised by NH). Coded data were reviewed by the research team. Codes were initially separated into groupings according to whether data were collected via focus groups or phone interviews to determine whether the different methods used were affecting the data collected. No differences in data were detected by the research team and subsequent analyses used combined data; however, carers in the individual phone interviews provided more in‐depth descriptions of their experience. Findings from carers were further analysed according to carer status as current or previous carers (by NH); no differences were noted by the research team, and data were combined. The coded data were then analysed using deductive methods of categorising and inductive methods to identify themes (analysis by NH and MB). Data were sorted and stored using Nvivo software and Microsoft Excel.

Demographic characteristics were summarised using descriptive statistics and analysed using SPSS software.

### 4.1 Findings

Two focus groups were conducted with 12 carers and 2 focus groups with 11 nurses, and an additional 33 carers and 4 nurses participated in phone interviews. Each focus group comprised 4 to 8 participants. Duration ranged from 74 to 80 minutes for carers and 30 to 55 minutes for nurses. Duration of phone interviews ranged from 20 to 73 minutes for carers and 13 to 56 minutes for nurses.

The majority of carers were female (n = 27, 60%) and were currently looking after someone with cancer (n = 35, 78%). The average age was 55 (SD: 14) years. Carers were mostly caring for a spouse or partner (n = 29, 64.4%), parents (n = 13, 28.9%), children (n = 1, 2.2%), friends (n = 1, 2.2%), or other relatives (n = 1, 2.2%). Most carers lived with the person undergoing cancer treatment (n = 39, 86.7%). The highest levels of education of carers were university degree (n = 21, 46.7%), high school (n = 9, 20%), certificate or diploma (n = 7, 15.6%), or other (n = 7, 15.5%). The majority of nurses were female (n = 10, 66.6%).

Reasons for not participating included: unable to be contacted (n = 24, 28%), too busy (n = 19, 22%), not being able to leave patients unattended (n = 12, 14%), not interested (n = 12, 14%), not receiving consent forms (n = 10, 12%), no perceived needs (n = 7, 8%), and illness (n = 2, 2%).

## 5 INFORMAL CARERS' ACCESS TO SUPPORT MECHANISMS TO ADDRESS INFORMATION AND SUPPORT NEEDS

The ability to access information and support when carers needed it was vital in providing reassurance and assisting them to manage or appropriately escalate patients' care needs. Carers sought support through a variety of sources including healthcare professionals, information brochures, the internet, and through word of mouth from other carers. Participants reported experiencing a variety of barriers when accessing support.

The lack of immediate assistance from health services was considered problematic. Nurses acknowledged that carers had significant needs in relation to the knowledge required to provide ongoing care for patients over the trajectory of their illness and treatment; they also recognised that carers have “…limited to no idea of what is happening” (N2). Information and education are usually provided early in the treatment trajectory, and by the time patients started experiencing side effects of treatment or illness progression, there were few opportunities for nurses within the health services to provide further education to support carers' needs.

You do not want too much (information and support) right at the beginning... but then I feel like they almost fall off the radar a little bit unless something (a need) gets identified. *(N3)*

Carers were comfortable in raising patients' care needs with healthcare professionals and actively asked questions, but at times, the information received “was not quite as accurate as it should have been” (C10), “confused more than it helped” (C14), inadequate in that “they tend to tell you only what you need to know” (C16), or nonexistent “I mentioned it to the oncologist and he just seemed to ignore me” (C30).

Despite nurses emphasising the availability of telephone support, “we give them our contact number and ask them to call us” (N3), carers were hesitant to inconvenience staff with the many day‐to‐day uncertainties they experienced and decisions that had to be made.

Do I annoy the staff at the hospital… if I am being silly about it (an adverse side effect). *(C21)*

As a result of the limitations in information provided by healthcare professionals and carers' hesitancy to clarify symptoms as they arose, carers reported needing to find information on their own.

Some carers described the booklets developed by cancer organisations as useful in addressing the patients' needs and in supporting them during the caring period.

A lot of the booklets... have been pretty comprehensive so the information in those has been probably enough to get us through. *(C5)*

Others struggled with the volume of information provided in written form and did not find the information sufficiently specific to their situation.

You have to wade through a whole heap of specific information on cancers that do not reflect the situation you are going through. *(C8)*

(I wanted more information on) home remedies… through to when it is appropriate to take paracetamol. *(C3)*

Because of the inadequacies in timing, volume and source of information carers sought alternative resources on the Internet.

Essentially…we find out as we go along, we Google it. *(C11)*

When carers needed immediate assistance however, accessing information on the Internet this way was a barrier to its utility because of the amount and relevance of the information available and difficulty in locating the information required.

I mean the cancer council site (website), it is fabulous but you have to really sit and read it. *(Current carer focus group)*

Carers' ability to seek information online also relied on their knowledge of credible websites available. Many nurses stated that they provided education to carers about using locally based cancer service websites for information; however, they were reluctant to recommend other appropriate resources for carers to access.

Google is our enemy a lot of the time…for the most part it makes I think our job a lot harder, and it makes it harder for them (carers) because they are just hearing a lot of misinformation. *(N3)*

The majority of carers did not recall receiving local cancer service information from healthcare professionals during their caring period.

No they have not mentioned that at all (in response to healthcare professionals recommending reliable websites). *(C37)*

Barriers to information seeking at times left carers with insufficient knowledge to support them in managing the patient's symptoms and side effects on their own at home. Carers found sourcing information, reassurance and support from others, particularly carers and patients who shared a similar experience, was beneficial; however, these occurrences were likely to be incidental.

I was lucky that one of the people that I worked with her husband went through a similar type of chemotherapy, so I got a lot more useful information out of her. *(C14)*

## 6 HOW TECHNOLOGY CAN BRIDGE INFORMATION AND SUPPORT GAPS

Carers and oncology nurses felt that technology may be useful in bridging information gaps and providing carers with support about how to manage symptoms and side effects at home and when to escalate care. Carers also described technology as a way for them to receive current, relevant information to suit their situation.

Technology may provide carers with opportunities to seek support in situations where they are hesitant to disturb nursing staff.

If you had a (smartphone) app…that reinforces if something like that happens (an adverse event) no ifs, buts, or maybes, get your butt back to the hospital…I would have used an app in that situation…It would be easier to use a (smartphone) app rather than thinking do I annoy the staff at the hospital (when unsure if symptoms require medical attention). *(C21)*

Carers and nurses both suggested that technology may be suitable for intermediary support in identifying serious side effects and recommending treatment as needs arise at home. Intermediary support may overcome barriers related to seeking information that is not readily available or in a form that is unmanageable because of its volume or diversity because technology can provide information that can be delivered in real time.

*(In reference to information technology, it may provide)* basic symptom management like when you should be contacting the hospital on behalf of your loved one. *(Nurse focus group)*

Carers noted that technology may be an appropriate way to deliver information and at times may be more up to date compared to information booklets or information researched independently online by carers.

*(In reference to cancer information booklets)* Anything that's actually printed in paper is going to be out of date in six months' time… Something online or that's on a (smartphone) app, something that's modern and, and up to date is much more trustworthy. *(C32)*

Carers also described the value of information delivered through technology in allowing for capabilities to streamline searches and reduce the burden of finding relevant information.

If you are putting in (to a technology application) my person has liver cancer, then it can sort of target your little tree down that way rather than all of these other things that may not be relevant. *(Past carer focus group)*

## 7 DISCUSSION

Caring for someone with cancer is complex. Patients experience a myriad of side effects and symptoms. Informal carers are required to monitor and manage symptoms as they arise, with little to no training and as described in this paper often with inadequate information and support to do so.

Adequate information was pertinent to carers' perceived ability to provide care for someone with cancer and reduce unmet needs and uncertainty in the caring role; this is consistent with previous research.^7^, ^8^ The provision of adequate information was impacted on by barriers experienced by nursing staff and carers such as the timing, source, and reliability of information. Both carers and healthcare professionals noted that the provision of information was inadequate and that carers continued to show uncertainty in managing symptoms throughout the illness trajectory. Current methods routinely provide carers with information only during the diagnosis phase, and there is little follow‐up on their needs.^8^

Discrepancies between carers' and nurses' responses in the current study make it difficult to conclude whether carers were informed of appropriate websites and support services to access. Previous studies have shown that healthcare providers can be instrumental in encouraging carers to assess support through technology, but that many carers are not recommended to do so.^12^ It is therefore important that healthcare professionals are well informed about existing resources and are confident in their provision of reputable information to enable them to alert carers of support systems available to them.

Quicker access to care services has been enabled through cancer‐based telephone services by successfully providing assistance and reassurance, information, and support.^12^, ^13^ Web‐based interventions also have the potential to improve communication with healthcare teams^14^ and enable carers to access support from other carers.^15^

Development of a technology‐based interface such as a smartphone application may provide carers with information and support specific to their needs. Regular updating and monitoring of information would be required to ensure that the content remained current. Ogah and Wassersug^16^ evaluated online information related to prostate cancer and found that after 40 hours of researching, only 43 out of 21 million websites provided current, relevant, and easy to read information. Further, the use of a single interface combining all information and support available to carers may reduce the time burden spent searching numerous existing resources. A smartphone application may improve healthcare professionals' trust in recommending its use if developed and supported by reputable sources and reduce staff burden in having to monitor and recommend numerous websites.

The potential usefulness of mobile technology in the provision of information and support to carers requires further investigation. Resources to meet the needs of people living in rural and remote locations or carers for whom English is not their primary language also requires investigation as these population groups may have their own unique needs.

## 8 LIMITATIONS

Study limitations need to be considered when interpreting these results. Carers from culturally and linguistically diverse backgrounds who did not have sufficient English language skills were excluded from the study. Carers who speak languages other than English may experience needs not identified in this study. Further, participants were recruited from a similar geographical location in Melbourne Australia, which may limit the generalisability of our findings to carers in regional and rural groups.

## 9 CLINICAL IMPLICATIONS

Carers of patients with cancer experience unmet needs for information and support in the management of day‐to‐day activities. Smartphone technology may represent a suitable platform in assisting carers. Researchers, health professionals, carers, and software developers should work together to develop a suitable smartphone application that reflects carer needs to support them in their role throughout the cancer trajectory, from diagnosis through to survivorship, palliative care, or bereavement.

## CONFLICT OF INTEREST

The authors declare no conflicts of interest.
